# Diurnal Rhythm of Plasma Melatonin Concentration in the Domestic Turkey and Its Regulation by Light and Endogenous Oscillators

**DOI:** 10.3390/ani10040678

**Published:** 2020-04-13

**Authors:** Magdalena Prusik, Bogdan Lewczuk

**Affiliations:** Department of Histology and Embryology, Faculty of Veterinary Medicine, University of Warmia and Mazury in Olsztyn, Oczapowskiego Str. 13, 10-719 Olsztyn, Poland; lewczukb@uwm.edu.pl

**Keywords:** domestic turkey, pineal, melatonin, plasma, rhythm

## Abstract

**Simple Summary:**

Environmental light regulates a wide range of phenomena in almost all organisms on Earth. Daily and seasonal changes in the photoperiod duration are the most important factors controlling the secretion of melatonin (MLT), a pineal hormone that affects many physiological processes in birds. The results of previous studies on the effect of MLT on the productivity and health of poultry have been promising. However, there are very few studies on the daily profiles of plasma MLT concentrations in domestic birds; therefore, we decided to examine plasma MLT levels in 10-week-old domestic turkeys exposed to different light conditions. The results demonstrated that plasma MLT concentration in turkeys kept under a 12 h light: 12 h dark cycle showed a prominent diurnal rhythm. Night-time light exposure caused a rapid decrease in plasma MLT concentrations. The housing of turkeys in continuous dim red light revealed endogenously generated diurnal rhythm of MLT secretion. The rhythm of the plasma MLT level in a reversed cycle of 12 h dark: 12 h light adapted quickly to the new lighting condition.

**Abstract:**

The aim of this study was to characterize the diurnal rhythm of plasma melatonin (MLT) concentration and its regulation by light and endogenous oscillators in 10-week-old domestic turkeys. Three experiments were performed to examine (i) the course of daily changes in plasma MLT concentration in turkeys kept under a 12 h light: 12 h dark (12L:12D) cycle; (ii) the influence of night-time light exposure lasting 0.5, 1, 2, or 3 h on the plasma MLT level; and (iii) the occurrence of circadian fluctuations in plasma MLT levels in birds kept under continuous dim red light and the ability of turkeys to adapt their pineal secretory activity to a reversed light-dark cycle (12D:12L). The plasma MLT concentration was measured with a direct radioimmunoassay. The plasma MLT concentration in turkeys kept under a 12L:12D cycle changed significantly in a daily rhythm. It was low during the photophase and increased stepwise after the onset of darkness to achieve the maximal level in the middle of the scotophase. Next, it decreased during the second half of the night. The difference between the lowest level of MLT and the highest level was approximately 18-fold. The exposure of turkeys to light during the scotophase caused a rapid, large decrease in plasma MLT concentration. The plasma MLT concentration decreased approximately 3- and 10-fold after 0.5 and 1 h of light exposure, respectively, and reached the day-time level after 2 h of exposure. In turkeys kept under continuous darkness, the plasma MLT level was approximately 2.5-fold higher at 02:00 h than at 14:00 h. In birds kept under 12D:12L, the plasma MLT level was significantly higher at 14:00 h than at 02:00 h. The results showed that plasma MLT concentrations in 10-week-old turkeys have a prominent diurnal rhythm, which is endogenously generated and strongly influenced by environmental light.

## 1. Introduction

Environmental light regulates a wide range of physiological processes in almost all organisms on Earth. The daily and seasonal changes in the photoperiod duration are the most important factors controlling melatonin (MLT) secretion by the pineal gland. Day-night rhythmic MLT secretion regulates in birds, among others, the diurnal rhythm of locomotor activity and feed intake [1,2,3,4]; body weight [2]; reproductive system functions [2,5,6,7,8]; immune system activity [9,10,11,12]; seasonal singing, migration, and spatial orientation during flight [8,13]; thermal homeostasis [14,15,16]; and energy metabolism [1,17]. MLT is also an important component of the antioxidative defense system [18]. The influence of the pineal organ and its hormone on the productivity and health of poultry has been previously studied, mainly for chickens and turkeys, with promising results. MLT supplementation and/or properly set lighting schedules resulted in a reduction in the incidence of sudden death syndrome [19], activity-related heat production [16,17], and isolation distress [20], in chickens. The better use of feed [2], the influence on growth of the ovaries and egg production [2,5], the improvement of male reproduction [21], and the acceleration of development of the cellular and humoral immune responses [10,11] have been described as the effects of such treatment in turkeys.

In the face of a growing number of studies proving the significant role of the pineal gland and its chief hormone in poultry farming, there are very few studies focusing on the profiles of plasma MLT concentration in domestic birds [18,22,23,24,25,26,27,28]. In most cases, the published data involve very young birds aged 2–4 weeks. Liou et al. [24] described significant differences in the nocturnal patterns of plasma MLT between chicks and laying hens. The peak of plasma MLT in laying chicken was broader than that in chicks, which was considered to be essential for the regulation of oviposition. 

The aim of our study was to determine in 10-week-old turkeys: 1) the course of the diurnal rhythm of plasma MLT concentration under a 12 h light: 12 h dark (12L:12D) cycle; 2) the influence of night-time light exposure on the plasma MLT level; 3) the occurrence of circadian fluctuations in plasma MLT levels in birds kept under a continuous dim red light; and 4) the ability of turkeys to adapt their pineal secretory activity to a reversed light-dark cycle. The data presented in this article show that the domestic turkey distinguishes among poultry species by the very high-amplitude of the diurnal rhythm of plasma MLT level.

## 2. Materials and Methods 

### 2.1. Chemicals

Antimelatonin antibody R/R/19540-16876 was provided by Dr. Jean-Pierre Ravault (Institut National De La Recherche Agronomique, France). 2-[^125^I]-iodomelatonin was purchased from Perkin Elmer (USA), gelatin was obtained from Merck (Germany), and the other reagents were obtained from Sigma (USA).

### 2.2. Animals and Materials

Female turkeys (*Meleagris gallopavo gallopavo var. domesticus*) were kept under a cycle of 12-h photophase (from 07:00 h to 19:00 h) and 12-h scotophase, starting from the 6th week of their postembryonic life. During the photophase, full-spectrum fluorescent lamps provided light with an intensity of 100 lx at the floor level and during the scotophase the turkeys were kept in red light with an intensity of 3 lx. Dim red light was used as an alternative for darkness, because it enables one to perform animal euthanasia and blood sample collection during scotophase without changes in light condition. Moreover, dim red light was necessary for maintenance of animals kept in continuous darkness in Experiment III. The animals had free access to standard food and water. 

### 2.3. Experiments

Three experiments were performed to determine the diurnal profile of the plasma MLT concentration in turkeys kept under a 12L:12D cycle (Experiment I), changes in the plasma MLT level in response to light exposure at night (Experiment II), and the circadian or diurnal variations in the hormone levels in the plasma of turkeys kept in continuous darkness or under a reversed dark-light cycle (12D:12L) (Experiment III). All experimental procedures on animals were performed in accordance with Polish and European Union laws (Approval of the Local Ethics Committee in Olsztyn for project KBN 6 PO6K 023 21). 

#### 2.3.1. Experiment I 

The experiment was performed in two replicates. Forty-two turkeys reared under a 12L:12D cycle between the ages of 6 and 10 weeks were used in each replicate. The birds were euthanized at 08:00, 10:00, 12:00, 14:00, 16:00, and 18:00 h (three individuals per time-point, n = 6 from two replicates) and at 20:00, 22:00, 24:00, 02:00, 04:00, and 06:00 h (four individuals per time-point, n = 8 from two replicates). Blood samples were collected to measure the plasma MLT concentrations. Plasma samples were frozen at −20 °C until subsequent MLT assay.

#### 2.3.2. Experiment II 

The experiment was performed in two replicates on 24 turkeys in each replicate. Ten-week old birds were divided into two equal groups: control and experimental group. At 01:00 h, the birds from the experimental group were exposed to fluorescent light with an intensity of 100 lx, while the control animals were kept under dim red light. Turkeys in both groups were killed at 01:30, 02:00, 03:00, and 04:00 h (three individuals per time-point from each group, n = 6 from two replicates), and blood samples were collected for MLT assay.

#### 2.3.3. Experiment III 

Three groups of birds, housed between 6 and 9 weeks of age under the 12L:12D cycle, were placed in separate rooms. During the next week, they were kept as follows: group I, in a normal light-dark cycle (12L:12D); group II, in a continuous dim red light with intensity below 3 lux (0L:24D); and group III, in a reversed dark-light cycle with a photophase from 19:00 h to 07:00 h and a scotophase from 07:00 h to 19:00h. After 7 days, the turkeys from each group were killed at 14:00 h and 02:00 h (five individuals per time-point from each group) and blood samples were collected. 

### 2.4. Melatonin Radioimmunoassay

MLT concentration in the plasma samples was measured by a direct radioimmunoassay [25,26,27,28,29] with rabbit R/R/19540-16876 antiserum [30] and ^125^I-melatonin used as a tracer. Antiserum R/R/19540-16876 (200 µL), diluted 1:15000 in assay buffer (tricine, 0.1 M; sodium chloride, 9 g/L; gelatin 1 g/L), was added to a 100 µL sample or standard (0–1000 pg/mL prepared in charcoal-stripped turkey plasma). The mixture was incubated at room temperature for 30 min and then 100 µL of ^125^I-melatonin, diluted in the assay buffer to approximately 10000 cpm in 0.1 mL, was added. After overnight incubation at 4 °C, the antibody-bound melatonin was separated from the free fraction by incubation with 250 µL dextran-coated charcoal (1.2 g Norit A and 60 mg dextran in 100 mL of assay buffer) for 15 min at 4 °C. After centrifugation (3000× *g*, 20 min at 4 °C), the radioactivity of 350 µL of supernatant was measured using the liquid scintillation method. The concentration of MLT in samples was determined using ImmunoFit EIA/RIA ver. 3.0A software (Beckman, Pasadena, CA, USA). The sensitivity of the assay was 4 pg/mL. Intra- and interassay coefficients of variation were below 10%.

The assay was validated by running the samples containing different amounts of exogenous MLT and the night-time samples diluted with charcoal-stripped turkey plasma. MLT added to samples of turkey plasma was quantitatively recovered (97–105%, R = 0.998–0.999). The samples diluted with charcoal-stripped plasma gave a displacement parallel to that of the standard curve.

### 2.5. Statistical Analysis

The data were analyzed using a one-way analysis of variance followed by Duncan’s test (Experiment I) or by t-test (Experiments II and III) using the Statistica 10.0 (StatSoft, Tulsa, OK, USA) software program. A value of *p* ≤ 0.05 was considered significant. 

## 3. Results

### 3.1. Experiment I

**Figure 1:**
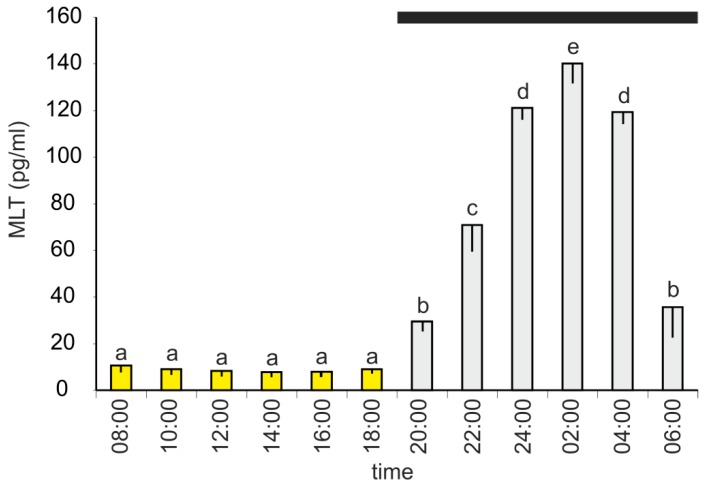
Experiment I. Concentration (mean, and standard error of the mean, SEM) of melatonin in plasma of 10-week-old turkeys kept under a 12L:12D cycle. Values flagged with different letters are significantly different. Black horizontal bar: period of darkness.

The plasma MLT concentration changed during a diurnal cycle and was significantly higher between 20:00 h and 06:00 h than between 08:00 h and 18:00 h (Figure 1). The lowest level of MLT (7.9 ± 1.9 pg/mL) was measured at 14:00 h; however, there were no significant differences between the investigated time-points of the photophase. The MLT concentration increased stepwise during the first half of the scotophase, reaching a maximum value of 145.1 ± 8.4 pg/mL at 02:00 h, and then decreased. At 06:00 h, the mean MLT level was approximately 3-fold lower than that at 04:00 h.

### 3.2. Experiment II

**Figure 2:**
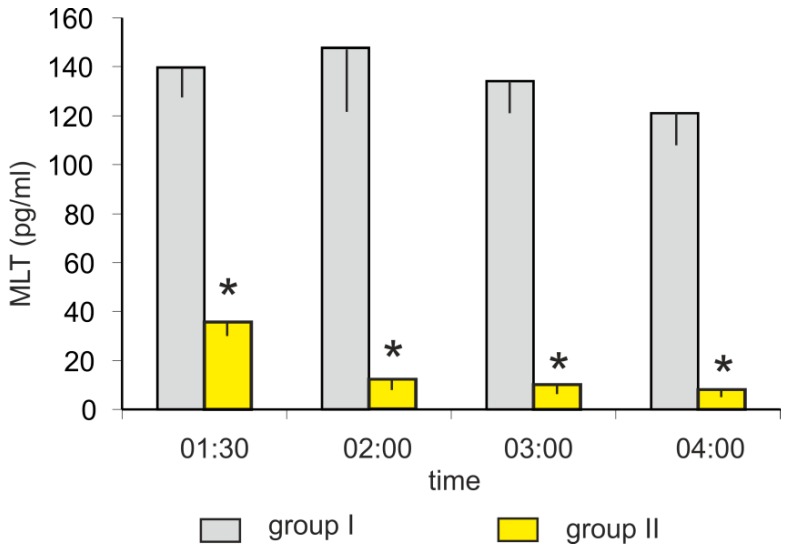
Experiment II. Concentration (mean and SEM) of melatonin in blood plasma in control (group I) and light-exposed (group II) turkeys at 01:30, 02:00. 03:00, and 04:00 h. The exposition of birds from group II started at 01:00 h. Values flagged with “*” differed significantly from the corresponding controls.

The mean plasma MLT concentrations in turkeys kept in darkness (group I) at 01:30, 02:00, 03:00, and 04:00 h varied between 120 and 150 pg/mL. The light exposure (group II) caused a rapid decrease in plasma MLT concentration (Figure 2). The MLT concentration was more than 3-fold lower after 30-min of light exposure (at 01:30 h) and approximately 10-fold lower after 60-min of light exposure (at 02:00 h) compared to the corresponding controls. The plasma MLT concentration after 2 and 3 h of light exposure decreased to approximately 10 pg/mL. 

### 3.3. Experiment III

**Figure 3:**
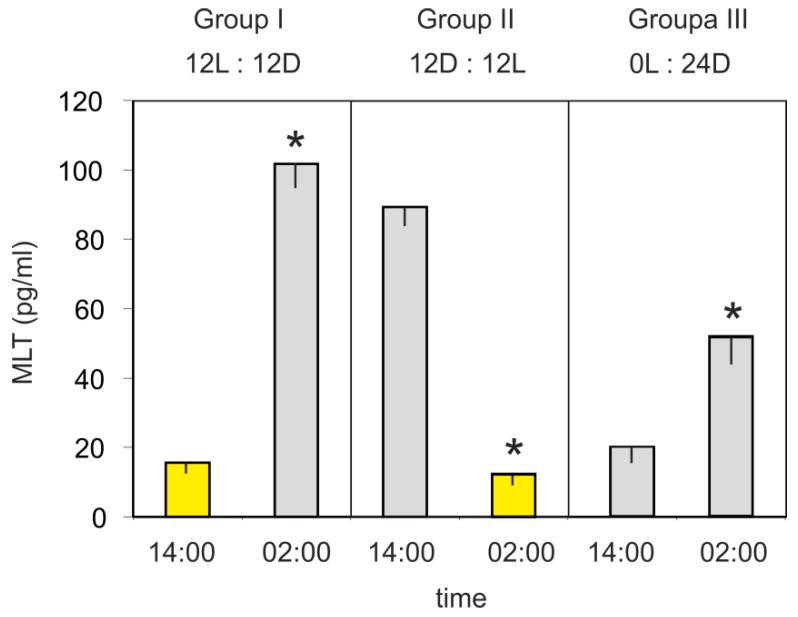
Experiment III. Concentration (mean and SEM) of melatonin in the blood plasma in 10-week-old turkeys kept under 12L:12D cycle (group I), 12D:12D cycle (group II), and continuous dim red light (group III) measured at 14:00 h and 02:00 h. Values significantly different between samples taken at 14:00 h and 02:00 h within each group were flagged with “*”.

The mean plasma MLT concentration in turkeys kept under a 12L:12D cycle (group I) was significantly higher at 02:00 h than at 14:00 h (Figure 3). In contrast, the hormone level was significantly higher at 14:00 h than at 02:00 h in the birds kept under a reversed 12D:12L cycle (group II). The mean plasma MLT concentration of turkeys kept in the continuous dim red light (group III) was significantly higher (approximately 2.5-fold) at 02:00 h than at 14:00 h.

## 4. Discussion

The obtained results showed that the plasma MLT concentration in 10-week-old turkeys, housed under a 12L:12D cycle, changed in a distinct diurnal rhythm. The MLT concentration was low during the photophase and increased stepwise after the onset of darkness to achieve a maximal level in the middle of the scotophase. Then, it decreased during the second half of the night. The profile of plasma MLT concentration in turkeys resembles type B of Reiter’s classification based on studies performed in mammals [31]. The maximal concentration of MLT, noted at 02:00 h, was approximately 18-fold higher than the minimal concentration at 14:00 h (Experiment I). The amplitude of day-night changes in plasma MLT concentration in 10-week-old turkeys was markedly higher than that in other domestic birds, especially when compared to chickens [18,22,24] and ducks [25]. Plasma MLT in chickens was 3–5 fold higher at night than during the day [18,22,24]. It is worth to note that both species—the turkey and the chicken—belong to the same avian family. The amplitude of day-night changes in plasma MLT in ducks reported by Zawilska et al. [25] was 11-fold. 

Our results differ slightly from those reported by Zawilska et al. [27,28] in younger turkeys, in which MLT concentrations measured during both photophase and scotophase were higher and the ratio between the minimum and maximum MLT concentrations was lower than in the present study. These differences suggest that the diurnal profile of plasma MLT concentration undergoes changes during the postembryonic development of the turkey. 

The daily profile of plasma MLT concentration described in the present study was similar to the diurnal cycle of MLT secretion in previous in vitro studies that were performed on the pineal organs of turkeys at a similar age [32,33]. However, the ratio between the maximum and minimum concentrations of MLT in the plasma was significantly lower than the amplitude of the MLT rhythm reported in the superfusion culture (18-fold in vivo vs. 40-fold in vitro). This difference may have several sources. First, the MLT level in the plasma is not only a result of the secretory activity of the pineal organ but is also, to some extent, a result of the absorption of MLT into the blood stream from the gastrointestinal tract [34,35] or other sites including the retina [36]. A significant share of the pool of the hormone circulating in the blood during the day-time may have MLT adsorbed from the seeds of cereals that are the main component of the feed for turkeys [37,38]. Studies performed on chickens and pigs revealed that MLT from the feed significantly affected the plasma levels of the hormone during the day [34,35]. In turn, the increase in secretory activity of the pineal organ at night makes the percentage of MLT originating from nonpineal sources very small. Second, plasma MLT concentrations are influenced by the intensity of the metabolism of this hormone in the liver [39,40]. Third, the pineal organ in culture is deprived of neuronal and hormonal regulatory factors and its activity may also be affected by artificial in vitro conditions. 

The exposure of turkeys to light during the scotophase caused a rapid decrease in plasma MLT concentration. Our study revealed that 30 and 60 min after light exposure, the plasma MLT concentrations were less than 4 and 1.5 times higher than the level occurring during the day, respectively. Two-hour exposure to light caused a decrease in the plasma MLT concentration close to the day-time level. The light-evoked decrease in vivo was significantly higher than that observed in similar in vitro experiments [32,33]. Results of the in vitro studies showed that even 3-h exposure to light did not decrease the MLT secretion to the level characteristic of the light phase of the cycle. The obtained data strongly suggested that light affects the pineal gland in two ways: directly on photosensitive pinealocytes and indirectly through the retina and adrenergic innervation. The stronger inhibitory effect of light in the in vivo experiments than in the in vitro experiments was probably the result of the inhibitory effect of norepinephrine released from the sympathetic fibers during light exposure. This explanation is supported by the data from in vitro experiments showing a stronger effect of norepinephrine than light on MLT secretion during the scotophase [32]. 

The experiment that housed the birds under continuous dim red light showed that the rhythm of plasma MLT concentration in the domestic turkey was generated endogenously. After 7 days in such conditions, the concentration of this hormone was still approximately 2.5-fold higher during the subjective night than during the subjective day. The lower amplitude of circadian fluctuations of plasma MLT concentrations resulted both from higher levels during the day-time and lower levels during the night-time. Similar data were obtained from younger turkeys, in which the circadian rhythm of MLT secretion persisted for 7 days under continuous dim red light and had an amplitude that was 50–80% lower than the rhythm in the 12L:12D cycle [27,41]. The circadian rhythm of MLT secretion with gradually decreasing amplitude was also observed in the superfusion culture of the turkey pineal organs for 5 days in continuous darkness [33]. However, the amplitude of this rhythm was very low starting from the third day of culture. The longer persistence of circadian MLT rhythm in vivo than in vitro suggests that the circadian oscillators that are located outside the pineal organ, that is, in the suprachiasmatic nucleus and retina, retain their activity without external photic time cues for a longer period than the pineal oscillator and influence pineal activity. In hens kept under continuous darkness, the circadian rhythm of plasma MLT persisted for 3 days without a decrease in the amplitude [22]. However, it decreased quickly in birds, in which sympathetic innervation of the pineal gland was interrupted [22,42]. In the superfusion culture, the circadian rhythm of MLT secretion persisted longer in the experiments with the turkey pineal organs than the chicken pineal organ [22,32,33,43]. In contrast, the circadian rhythm of MLT secretion from the pineal glands of Japanese quail cultured in constant darkness was very weak or completely abolished [44]. However, the Japanese quail is the only known exception among birds investigated to date in which the pineal oscillator does not play a significant role in the circadian pacemaking system [45]. It is worth noting that circadian rhythms in birds are controlled not only by light but also by other factors acting especially on peripheral oscillators located in other sites than pineal gland, the retina, and the suprachiasmatic nucleus. For example, it has been proven that different feeding regimes significantly affected mRNA expression of circadian clock genes in the liver, jejunum, and kidney [46,47,48]. 

After 7 days of housing of 10-week-old turkeys under a reversed dark-light cycle (12D:12L), the diurnal rhythm of the plasma MLT concentration was adapted to the new lighting conditions. In an in vitro experiment, turkey pinealocytes entrained their secretory activity to such changes on the second day of culture [32,33]. The adaptation of the chicken pinealocytes to the 12D:12L cycle was observed on the third day of culture [43]. 

Our results showed that the turkey pineal organ not only secrets MLT in a daily rhythm with very high amplitude but also responds quickly and precisely to changes in light conditions. These findings have important practical aspects, considering the significance of the domestic turkey as meat-producing animal. Schwean-Lardner et al. [49] exposed turkeys to different cycles with varying periods of darkness and studied body weight, feed efficiency, skeletal disorders, mortality, mobility, and ocular measures, in birds. Most of these factors achieved their best results in cycles with the shortest days. Only feed efficiency was higher in cycles with longer days. MLT extends many variable effects on the organism, which are related to the wide distribution of its receptors in the brain and other organs: the heart, arteries, adrenal gland, kidney, lung, liver, gallbladder, small intestine, adipocytes, ovaries, uterus, breast, prostate, and skin [50]. The pineal hormone may also act intracellularly by binding to calmodulin and Z retinoid nuclear receptors. MLT is one of the most powerful natural antioxidants acting by direct chelation of oxygen and nitrogen reactive species and by mobilization of the intracellular antioxidant enzymatic system [50].

## 5. Conclusions

In summary, in the present study, we found that 10-week-old turkeys manifest the prominent diurnal rhythm of the plasma MLT concentration, which is endogenously generated and strongly controlled by light. Turkeys are thus a very good avian model for further in vivo studies on the mechanisms regulating MLT secretion from the pineal gland.
